# A Proposed Probabilistic Method for Distinguishing Between Delusions and Other Environmental Judgements, With Applications to Psychotherapy

**DOI:** 10.3389/fpsyg.2021.674108

**Published:** 2021-08-09

**Authors:** Boopala Arul, Daniel Lee, Sarah Marzen

**Affiliations:** ^1^Division of Biological Sciences, University of California, San Diego, San Diego, CA, United States; ^2^W. M. Keck Science Department, Pitzer, Scripps, and Claremont McKenna College, Claremont, CA, United States

**Keywords:** schizophrenia, hallucinations, delusions, Bayesian probability, cognitive behavioral therapy, reality testing

## Abstract

How can individuals with schizophrenia best be equipped to distinguish delusions from accurate judgements about their environment? This study presents an approach based on the principles of Bayesian probability and presents the results of a series of tests in which a simulated observer classifies randomly generated data characteristic of a simulated environment. The complexity of the data ranges from scalars to vectors of variable lengths, and the simulated observer makes its decisions based on either perfect or imperfect models of its environment. We find that when a low-dimensional observation is considered characteristic of both real observations and delusions, the prior probabilities of any observation being real or fake are of greater importance to the final decision than the attributes of the observation. However, when an observation is high-dimensional (complex), classification accuracy tends to improve toward 100% with increasing complexity of observations, as long as the patient's model of the world isn't drastically inaccurate. On the contrary, when the observer's model is sufficiently inaccurate, the accuracy rate decreases with increasing observational complexity. Overall, the results suggest applicability of the Bayesian model to the use of interventional therapy for those who suffer from psychosis.

## 1. Introduction

The American Psychiatric Association's DSM-5 (2013) categorizes schizophrenia as a spectrum of psychotic disorders whose characteristic symptoms include delusional beliefs and hallucinatory sensations which impair ordinary thinking and behavior, and lead to dysfunction in social and occupational life (American Psychiatric Association, 2013).

Factor analysis has, since the late 1980s, identified three general categories of symptoms; the symptoms associated with one factor are more likely to occur alongside each other than with the symptoms associated with other factors (Liddle, 1987).

The “positive symptoms” made famous by their association with florid states of psychosis, such as hallucinations and delusions, are only one such group. The second group, the negative symptoms, may include a flattening of speech and gestures that drastically reduces emotional expressiveness, or decreases in spontaneous movement to the point of catatonia.The third factor, meanwhile, is associated with a lack of coherence or abstraction in thought.

This study focuses on the problem of separating delusions from reality, but will first consider what phenomena may reliably be named as delusions, how these phenomena differ from each other, how they interact with each other and with phenomena outside the group, and which categories may be amenable to our approach.

Delusions, like hallucinations, are a positive symptom of schizophrenia—an addition to the lived experience absent from the experiences of those without schizophrenia. Delusions and hallucinations may both also occur in affective disorders, which include bipolar disorder, major depression, and post-traumatic stress disorder. A detail that might confirm a diagnosis of schizophrenia would be the extension of a plausible idea into absurdity; a patient might have sense of being carefully watched by others or being addressed with messages specifically for them, which becomes a delusion when that person is convinced that obviously disinterested actors like newspapers or TV shows, which are meant for a mass audience, are specifically referring to them and alluding to their circumstances (McKenna, 2017). In this way a delusion can assimilate anything in a person's perception, assigning motivations and responsibilities— but the main character of a delusion is usually the person holding the belief. Situations seem to them to be created with them in mind; people around them are talking to each other about them, or organizing something with them at the center (McKenna, 2017).

Where do the delusions of schizophrenia come from, and what strengthens them? A state of “delusional mood” has been described in some people, in which a familiar environment feels odd, uncanny, or different in some indescribable way, and events in that space are assigned new meaning or significance. A person may consider delusional-sounding explanations at this stage and reject them, or have them crystallize into deeply held ideas (McKenna, 2017). Similar descriptions have been given of auditory verbal hallucinations (AVH), or “hearing voices”: individuals with schizophrenia distinguish between their own “inner speech” (the phenomenon of “thinking to yourself” or imagining your thoughts as a voice) and AVH on the basis that they feel no ipseity (the quality of being created by and belonging to oneself, also described as “authorship” or “mineness”) toward their AVH. Something that should be familiar to the subject isn't familiar anymore, and they ascribe some other meaning or identity to what they cannot attribute to themselves (Rosen et al., 2018). Some delusional patients may proceed in this way to read meanings into the arrangement or other qualities of inanimate objects (a delusion of reference), or read a hidden and deeply personal meaning into the mundane interactions of other people with each other or with the patient (a delusion of reference). However, delusions of reference or misinterpretation are not always preceded by delusional mood (McKenna, 2017). Furthermore, a delusion doesn't have to stem from or be centered around an external event. Instead, delusions can, starting from a thought or some other internal event like a hallucination, still develop into a deeply held opinion, proposition, or judgement on oneself or one's status. To summarize, a delusion begins with a snap judgement on an object of personal significance, which is elaborated on and pushed into an absurd direction by further snap judgements.

The delusions of schizophrenia have some overlap with those seen in other disorders, or with non-delusional forms of strong or overvalued beliefs. However, the delusions of other disorders can be different enough in their onset and development to warrant different therapeutic assumptions and practices. Paranoia or “delusional disorder” distorts ideas in a manner similar to schizophrenia, seeking references from the environment or characteristics of oneself on which some opinion can be built. Persecutory variants lead to feelings of danger from others, and grandiose variants lead to feelings of superiority over others. A fifth of patients diagnosed with delusional disorder go on to be rediagnosed with schizophrenia. However, the peak age of onset for delusional disorder is the late thirties or mid forties, while the peak age of onset for schizophrenia is late adolescence (Waters and Fernyhough, 2016), and 60–80% of patients diagnosed with delusional disorder are married (McKenna, 2017). Prodromal schizophrenia may become fully developed through a period of stress, but delusional disorder is associated with the stresses and anxieties of a very different stage of life. The delusions of schizophrenia especially should not be conflated with those of affective disorders, like bipolar disorder, major depression, and post-traumatic stress disorder (PTSD). “Depressive cognitions” may include low self-regard, considering oneself unequal to one's responsibilities, and wishing to escape; in some cases of fully delusional thinking, patients may hold themselves responsible for inexplicable or supernatural disasters affecting people they care about (McKenna, 2017). Delusional thinking in affective disorders draws on affect or mood, integrating ideas of personal guilt, sin, and vulnerability that schizophrenia may not. The remainder of the study will discuss therapies optimized for schizophrenia, and the approach promoted by this study will only tangentially focus on the emotive content of the patient's observations or the patient's own mood at the time of making them. Our approach will therefore also be evaluated with respect to its possible effectiveness specifically against schizophrenia.

First developed in the 1990s, cognitive-behavioral therapy (CBT) is a prominently used family of therapy methods for several disorders, sometimes used along with antipsychotic medication (Lencer et al., 2011). As a treatment, CBT for psychosis (CBTp) embraces a more reactive approach, focusing on reducing the emotional distress associated with the positive symptoms of schizophrenia, developing new coping strategies, and shifting established ways of thinking about one's own symptoms (Hagen et al., 2011). Recently, CBT has had some new developments, sometimes referred to as third wave therapies, including compassionate mind training, eye movement desensitization, and reprocessing (Sommer et al., 2012). We believe our study supports another “third wave therapy,” that utilizes Bayesian probability to teach patients to learn the algorithm implemented in this study.

This study is a preliminary exploration of the possibility and promise of treating the separation of delusional judgements on the environment from accurate observations as a problem of probability. We propose an approach for separating real sensory perceptions from delusions that focuses on identifying and building, through collaboration between individuals and their therapists, two personalized distributions of attributes which the patient considers typical of an accurate observation about their environment, and typical of their conserved delusional thinking, respectively. The question of what an “accurate” observation is might be addressed in sessions between patients and therapists that draw on CBT methods like reality testing, and identification of the common traits underpinning the patients' delusions could benefit from metacognitive examination of the feelings that underpin that range of thinking. The question of whether a particular thought about the environment is reliable or not can then be expressed by the patient as a question of whether the attributes of that thought are more representative of the accurate or delusional distribution, or whether the thought is more likely to have arisen from one distribution or another given the nature of its attributes. This is suited for a direct application of, or else a style of thinking modeled on, Bayes' rule of conditional probabilities, which will be discussed in the next section.

To summarize, the patient builds off initial assumptions of what's “weird” or “strange” to build a more detailed description of where “strange” begins and ends, where the “normal” or “expected” begins and ends, and where the two might overlap. In the last phase the patient, unaided by a second opinion but assisted by their constructed model, identifies their thoughts on the environment as accurate or delusional.

**Figure 1:**
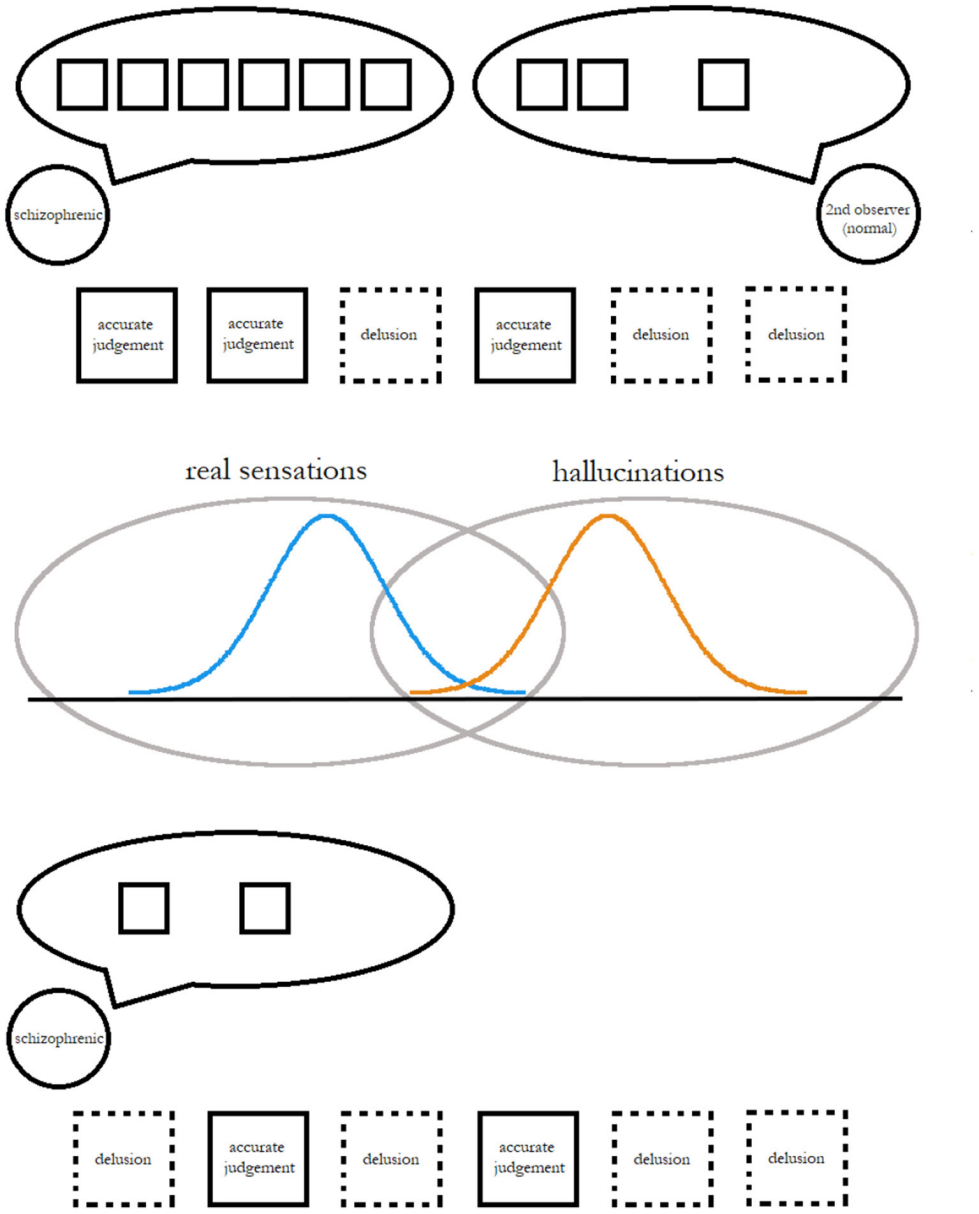
A simplified depiction of a proposed scheme to measure and improve the effectiveness of individuals' cognitive techniques for determining the reality of an observation.

Implementing this idea in a randomized controlled trial, or even a pilot study, is outside the scope of this paper. We consider ourselves to be establishing the groundwork for a pilot study by (1) working out a theoretical basis for our approach, (2) suggesting methodologies that could be used by a study with human subjects, and (3) testing a computer simulation of our approach and analysis of results. First, we continue the investigation of the literature begun in this section in section 2, to verify our theoretical assumptions, identify a subset of individuals with schizophrenia that may be well-suited for our approach, and lay out the problem in more detail. In particular, section 2.1 will describe the history of similarly-structured experiments in psychological research, and the dominant conclusions from them. Second, section 2.2 will show how virtual reality (VR) technology could contribute to implementing the system of controlled observation and categorization of delusions described in Figure 1. Third, we conduct several idealized simulations, with varying initial parameters, of an observer using Bayes' theorem to discriminate between two observational distributions. Instead of complex clustering approaches, we have chosen a more simple discrimination method that may be imitable by humans with minimal training. In fact, some believe that humans are Bayesian and can manipulate probabilities in a way that is at least somewhat logical (Griffiths and Tenenbaum, 2007). The ultimate goal of our approach is to build in patients with schizophrenia an appreciation and active use of the basic assumptions of Bayes' rule, and the proximate goal of the simulation would be to set an upper bound for the effectiveness of this minimalist approach, or to determine the highest extent to which we can expect this approach to work. The results of this simulation and immediate conclusions from them are discussed in Section 4.

We hypothesize that training people with schizophrenia to represent the reality of their observations perspective, and then to decide an observation based on those probabilities, has enough potential for therapeutic benefit to warrant further study. The strengths and weaknesses of our investigation, and the ultimate validity of our hypothesis, will be discussed in Sections 5 and 6.

## 2. Background

In this paper, we are validating a therapeutic method that requires individuals with schizophrenia to manipulate probabilities. In this section, we describe evidence that such an approach might succeed.

### 2.1. Schizophrenia and Probability Evaluation

In what follows, we will describe a proposed therapy that relies on individuals with schizophrenia correctly evaluating probabilities. It may be hard to imagine a human juggling high-dimensional vectors and precisely calculating the likelihood of each component. However, a simpler version of the same task, with simple proportions and smaller vector sizes, has for decades been a recurrent tool in examinations of the effects of schizophrenia on reasoning.

Preliminary studies by Hemsley and Garety (1986), Fischhoff and Beyth-Marom (1983), and Volans (1976) inspired the deployment of the “beads task” by Huq et al. (1988). This team tested the performance of 15 patients with active delusions, 15 patients with disorders other than schizophrenia, and 15 controls. In the beads task, the patient is informed that the test will involve two jars of beads, which both contain beads of two different colors. The jars have different ratios of beads: one might have a 80:20 ratio of color A to color B, while the other has a 20:80 ratio. Harder versions of the task will make the jars less distinct (60:40 and 40:60 ratios) or make it so the jars' ratios aren't reciprocal. Beads are drawn from exactly one jar, and the resulting sequence of beads becomes the subject's means of determining which jar is the source of the beads. Variations of the task may include switching beads with emotionally significant items like positive or negative comments about people, but even these items are framed as being “drawn from jars,” where each jar represents a different ratio of the possible variants of items. In discriminating between two different distributions through sequences of observations and calculations of likelihood, our approach resembles the beads task. Our simulation of the beads task differs in one respect, however— in the simulation, the observations are not qualitative traits with stark differences (like bead color) but real numbers drawn from two continuous random variables.

Early studies tried to prove a “jumping to conclusions” (JTC) bias characteristic of delusional subjects, by arguing that a lower average “draws to decision” (number of beads a subject has to see being drawn before they are ready to decide which jar is the source) was associated with delusion presence or severity. However, these studies' conclusions were still expressed with reservations about whether the subjects had really understood the task, or if some other confounding variable was at play (Moritz and Woodward, 2005). Associations with clinical variables are only found on harder versions of the task; one version that used reciprocal 20:80 and 80:20 ratios found no significant correlation between delusion-measurement scales and draws to decision despite a sample size of 300 (Pytlik et al., 2020). Controlling for strength of negative symptoms and IQ the association with positive symptoms, and delusions in particular, becomes non-significant (McKenna, 2017). The connection with delusions is more tenuous when one considers that JTC measures do not improve in response to antipsychotic medication, which reduces delusion strength (So et al., 2015). Meanwhile, one recent study using a variant of the beads task counteracted lack of motivation or interest in the task by punishing hastily-made wrong decisions through deductions from a monetary reward, and found that the variable that JTC was mostly strongly correlated with was a non-clinical variable—namely, low socioeconomic status—while delusional patients actually showed increased draws-to-decision (Baker et al., 2019).

Reduced draws-to-decision now seems less like the delusional bias it was initially framed as, and more like a cognitive deficit that may only be weakly, if at all, related to delusions. Lunt et al. (2011) administered the beads task to 19 people with frontal lobe lesions, and found significantly reduced draws to decision. Another theory that fails for similar reasons as the idea of a delusional “jumping to conclusions” bias is the idea that changes in theory-of-mind are responsible for delusions. Theory of mind refers to the ability to infer the mental states of others, and one possible theory of delusions was that individuals with schizophrenia built and maintained delusions through misrepresentations of their own mental state (seeing their own inner speech as auditory hallucinations resembling the voices of others, or otherwise taking on a foreign or invasive quality) and the mental state of others (reading meaning into the meaningless, or a reference to oneself in the interactions of the disinterested). However, studies of low performance on theory-of-mind tests have generally shown stronger correlations with negative symptoms (flattened affect, etc.) and formal thought disorder/disorganization than with positive symptoms (McKenna, 2017).

Based on the evidence, it is unlikely that delusions lead to inevitable impairment in probabilistic reasoning, but it is probable that strong negative symptoms lead to the same. Our approach may, unfortunately, be totally unsuitable for patients with strong negative symptoms or cognitive deficits. Across the spectrum of possible presentations of schizophrenia, we may predict that our approach undergoes a reduction in effectiveness that scales with the strength of negative symptoms, basing this prediction off the association of negative symptoms with reduced draws-to-decision, which manifests in the simulation of our approach as a reduced observational vector size. We may predict that different patients will be able to go up to different vector sizes, isolating different numbers of components from their judgements before arriving at a final decision on how to categorize that judgement. Our simulation must therefore not only predict the maximum possible effectiveness of our approach, but must set the upper bound for effectiveness even for small, non-ideal (but perhaps more realistic) vector sizes.

Existing approaches to cognitive therapy integrate education about the nature of cognitive biases. The beads task could be integrated into our approach as a teaching tool, that introduces and reinforces principles of probability and use of Bayes rule. Another example task might include an accurate estimation of coincidence probability (Griffiths and Tenenbaum, 2007).

### 2.2. Measuring Delusions and Analyzing Measurements

The simple depiction of delusional ideation and discrimination of truth from delusion in Figure 1 may give some readers pause: if the patient is recalling memories of delusional judgements, how is the therapist (who may not have been around to see, for example, an arrangement of background objects or a bystander's attitude which the patient considers significant) supposed to prove or disprove these specific assessments, and how will the patient avoid recall bias? Even if the emphasis is on delusional judgements which the therapist or some other neutral party (a parent or friend) was around to see and independently evaluate, can these ideas really be classified on demand, especially within the short sessions (30–40 min) of CBT?

One approach that produces current and independently verifiable delusional judgements in a safe, reliable way may be virtual reality (VR). Over the last 10 years, since the introduction of the Oculus Rift and other consumer-friendly VR devices, a common experimental setup has involved setting up environments like train interiors or elevators, where a patient's avatar shares space with several other avatars. The situations are emotionally neutral, but may be environments in which the patient comes to feel the attention of many people on them and develops delusions of reference or paranoia (Brown et al., 2020). Such associations will be coincidental, since the environment has not been constructed to produce them; but coincidences are themselves phenomena in which a certain event seems to be more likely under an alternate theory or hypothesis than the currently-held one, inviting reconsideration of causal explanations (Griffiths and Tenenbaum, 2007). The embrace of this alternate theory is characteristic of the reasoning style associated with schizophrenia.

The experiences produced in virtual reality can be analyzed with the same measures used for asking people about real-life experiences. In one study, Freeman et al. use six different assessment scales measuring paranoid, affective, and other symptoms (Freeman et al., 2010). Another review of VR in psychological research makes note of the possibility of real-time physical measurement, with eye-tracking (useful for analyzing targets of the subject's gaze or eye contact, in studies on social skills), heart rate monitoring, and galvanic skin response (sweat changes the skin's electrical conductance, allowing detection of sweaty palms which may further indicate emotional unease) (Freeman et al., 2015).

This study makes no particular commitment to virtual reality, and only suggests it as a promising option. Its merits or drawbacks are further discussed in section 5.

## 3. Theoretical Framework

Relevant features of probability theory are reviewed in section 3.1. In section 3.2, we describe our model of the world and the observer.

### 3.1. Probabilities and Probability Density Functions

We will take a frequentist approach to describing random variables and probabilities, but will then rely heavily on Bayes theorem as our basis for how an observer might classify judgements as delusional or not. There is no internal contradiction here; frequentist and Bayesian arguments simply boil down to different but compatible interpretations of probabilities, as will be explained.

Although there are more formal ways of describing this, a random variable *X* takes on a realization x∈X in an “experiment” in which one of many possible realizations is randomly produced. Each possible value *x* has a particular probability *p*(*x*) of occurring; if one repeats the experiment a large number of times, then the frequency of *x*'s will be nearly *p*(*x*). One can also view *p*(*x*) as describing our belief about the likelihood of getting *x* in any particular experiment. The quantity *p*(*x*) is referred to as a probability distribution. For example, let *X* represent a coin flip. The realizations can take on one of two values, x∈X={H,T}. If the coin flip is fair, then $p\left(H\right)=p\left(T\right)=\frac{1}{2}$. When the set of possible values for the realization is finite, the random variable is said to be discrete, as in this coin flip.

**Figure 2:**
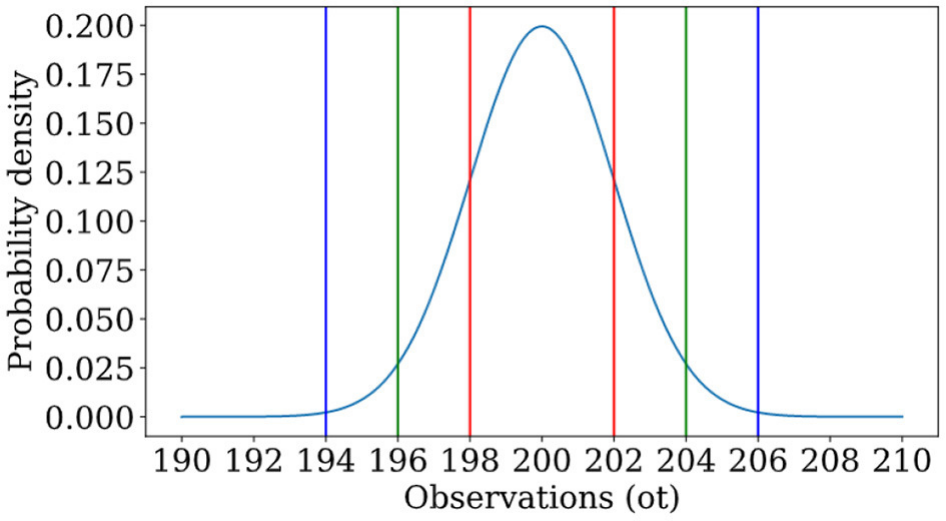
An example of a normal distribution, in which a random variable has probability density function $\rho \left(x\right)=\frac{1}{\sqrt{2\pi \sigma^{2}}}e^{-{\left(x-\mu \right)}^{2}/2\sigma^{2}}$ with μ = 200, σ = 2. One is most likely to see observations near the mean, and the likelihood of an observation is governed by how many standard deviations one is away from the mean. Lines indicate locations at which one is 1 (red), 2 (green), or 3 (blue) standard deviations from the mean.

When this is not the case, the random variable might be continuous (e.g., when X=ℜ) or mixed (a mixture of discrete and continuous). In this article, we will need to describe a continuous random variable *X* with realizations *x* in which $X={ℜ}^{n}$, an *n*-dimensional vector with real values, as this is how we will model sensory perceptions (imagine unfurling an *L* × *L* image into an *L*^2^ long vector of pixel intensities). In a great many experiments, we will never see the exact same vector twice; the frequency with which we observe the exact same vector in many experiments is exactly 0. However, we can ask about the probability (frequency) with which we observe a vector whose entries fall between two prescribed limits, written as *P*(*x* ≤ *X* ≤ *x* + 〈δ*x*_1_, …, δ*x*_*n*_〉). For small δ*x*_*i*_, this will take the form ρ(*x*)δ*x*_1_…δ*x*_*n*_. The quantity ρ(*x*) is referred to as the probability density function of the random variable *X*. A common example of such a probability density function is the normal distribution shown in Figure 2.

We are also interested in joint probabilities of two random variables *X, Y* and so-called conditional probabilities. Where two random variables are related (*X* may represent the result of a coin flip, while *Y* may represent the result of *m* rolls of a dice, where *m* depends on the results of the coin flip) we can ask: how does our observation of *Y* change based on knowledge of *X*, or vice versa? The answer is encapsulated in conditional probability distributions or densities *p*(*y*|*x*) and *p*(*x*|*y*). Simply put, these are the probabilities of observing *y* (or *x*) in an experiment given that we have observed *x* (or *y*).

### 3.2. Simulating World and Observer

The proposed method from Figure 1 outlines three phases of development. First, the patient works with a therapist who can affirm or challenge their judgements about their environment (VR environment or otherwise), or else devise a test of the assumption that may lead the patient to the therapist's intended conclusion. During this period of supervised observation, the patient acquires a sense of the prior frequency of delusion *f*′.

Concurrently, the patient constructs distributions of delusional and accurate judgements. The therapist may explain this process as “itemizing” one's views of the world, or making a checklist of essential qualities. Just as the beads task described in section 2.1 becomes easier to solve as more beads are drawn, the task of building a distribution of essential qualities for real and delusional observations or of their attitudes toward them, and later identifying an unknown observation as coming from either distribution, relies not only on making many observations but breaking down each one by prominent sensory details, emotive content, visible relations of cause and effect, implications, and other details on which a difference in distributions could be established. The patient may be encouraged to start with a short list, run through it a few times when in an unfamiliar situation, and then add onto it with time. The length of this list corresponds to the complexity of the task.

Then, the patient may begin to classify their own judgements in real time as accurate or delusional based on the estimated frequency of delusion and her estimations of how real and delusional observations are distributed. The simulation discussed below is an analogical representation of this process using generated observations and a simulated observer, which we have implemented in a computer program.

When generating observations or judgements *o*, we will err on the side of simplicity, though one could imagine the framework being applied to “records of thoughts” in the form of natural-language text samples such as patient interview responses. Each observation *o* will be an *n*-dimensional vector, where n has a minimum of 1. A 1-dimensional vector is a single number, and we can suppose this number will be drawn from a single number line representing all real numbers. The set of all real numbers is here used to analogically represent all the possible observations that a person can make about a specific feature or topic of their environment. In the same way that some observations are more likely than others, certain numbers are more likely to make up the observations *o*.

*T* observations will be generated, and each observation *o* will be an *n*-dimensional vector. Each component will be chosen independently of all others, but all components will be drawn from only one of two possible distributions. The *i*^*th*^ component *o*_*i*_ of any given judgement will be described by probability density function ρ_*r*_(*o*_*i*_) when the judgement is accurate and ρ_*f*_(*o*_*i*_) when the judgement is delusional. A biased coin flip with probability *f* of success (MacKay, 2003) determines whether or not the delusional distribution is chosen to generate an observation. All the trials used in our simulation will have the same ρ_*r*_(*o*_*i*_), but different means and standard deviations for ρ_*f*_(*o*_*i*_). This reflects the individualized nature of delusions, and the necessity of our approach being robust enough to deliver promising results under several possible scenarios.

The probability density function for the entire judgement *o* is $\prod_{i=1}^{n}\rho_{r/f}\left(o_{i}\right)$. Now, when receiving the judgements *o*, the observer models the probability density function of *o*_*i*_ as ${\tilde{\rho }}_{r/f}\left(o_{i}\right)$. The farther away $\tilde{\rho }$ is away from ρ, the worse the individual's model.

## 4. Results

In this section, we will turn to a simulated observer to get a sense of the maximum effectiveness of this approach for humans, and any possible limitations that are inherent to the approach.

The simulated observer described below can accommodate observations as described above. The observer's evaluation method is not tied to the environment's method for generating observations— although the two are related in design, the observer's “perception” of environmental parameters is allowed to differ from the “true values” used by the environment.

### 4.1. Classification of Observations

According to Bayes theorem, if we have two random variables *X* and *Y* with respective realizations *x* and *y*, then

(1) $$\begin{array}{r} p\left(y|x\right)=\frac{p\left(x|y\right)p\left(y\right)}{p\left(x\right)}. \end{array}$$

Bayes theorem in the context of cognition often interprets *Y* as hypothesis about the world and *X* as data. In this context, *p*(*y*) is our prior probability distribution over hypotheses, our subjective belief about how likely each world model is to be true. The conditional probability *p*(*x*|*y*) is the probability that some data *x* is produced if the world operates according to hypothesis *y*, often called the likelihood. Whereas, the conditional probability *p*(*y*|*x*) is the probability that hypothesis *y* is accurate given observed data *x*, also called the posterior. The evidence *p*(*x*) encapsulates the likelihood that such data occurred at all. One can therefore reasonably use Bayes theorem to update beliefs about the world given data. Acquisition of additional data can result in another Bayes update, using the previous posterior as the new prior.

The classification algorithm is not given confirmation as to whether each term is real or fake; its goal is to guess that information. It must estimate *p*(*R*|*o*_*t*_), the probability that *o*_*t*_ was drawn from ρ_*r*_ given its value, as well as *p*(*F*|*o*_*t*_), the probability that the term was drawn from ρ_*f*_ given its value. These come directly from Bayes theorem:

(2) $$\begin{array}{rcl} p\left(R/F|o_{t}\right) & = & \frac{p\left(o_{t}|R/F\right)p\left(R/F\right)}{p\left(o_{t}\right)}, \end{array}$$

where *p*(*R*) and *p*(*F*) are the prior probabilities that an *o*_*t*_ of any value was drawn from a particular distribution. To decide if an observation is accurate or delusional, we will simply take the ratio of *p*(*R*|*o*_*t*_) to *p*(*F*|*o*_*t*_). We call this ratio *L*:

$$\begin{array}{rcl} L & = & \frac{p\left(R|o_{t}\right)}{p\left(F|o_{t}\right)}=\frac{\frac{p\left(o_{t}|R\right)p\left(R\right)}{p\left(o_{t}\right)}}{\frac{p\left(o_{t}|F\right)p\left(F\right)}{p\left(o_{t}\right)}}=\frac{p\left(o_{t}|R\right)p\left(R\right)}{p\left(o_{t}|F\right)p\left(F\right)} \end{array}$$

(3) $$\begin{array}{rc} = & \frac{\rho_{r}\left(o_{t}\right)\left(1-f^{\prime }\right)}{\rho_{f}\left(o_{t}\right)f^{\prime }}, \end{array}$$

where the prior probability that any observation is delusional is thought to be *p*(*F*) = *f*′ and the probability that any observation is real is thought to be *p*(*R*) = 1−*f*′. Similarly, the probability (densities) of the observation is thought to be $\rho_{r/f}\left(o_{t}\right)=\prod_{i=1}^{n}{\tilde{\rho }}_{r/f}\left(o_{t,i}\right)$, as described earlier. Substituting these values gives

(4) $$\begin{array}{r} L=\frac{1-f^{\prime }}{f^{\prime }}\overset{n}{\underset{i=1}{\prod }}\frac{{\tilde{\rho }}_{r}\left(o_{t,i}\right)}{{\tilde{\rho }}_{f}\left(o_{t,i}\right)}. \end{array}$$

The classification algorithm need not exactly match the parameters used by the generative algorithm, and we discuss the implications of mismatch later on.

The following sections describe three sets of trials. Within each set, trials differ in parameters like the mean and standard deviation of the distribution from which delusional observations are drawn, to simulate specific and distinct tendencies under the general banner of “delusional ideation.” Trials may also differ in how accurate the observer's perception of the environment is. We will show that the observer is better at classifying complex observations than simple ones, and that this holds true even in select cases where the observer has an incorrect model of the environment, though not in others. In this latter category, a direct relation of observational complexity and classification accuracy fails to manifest.

### 4.2. Misclassification in One Dimension

**Figure 3:**
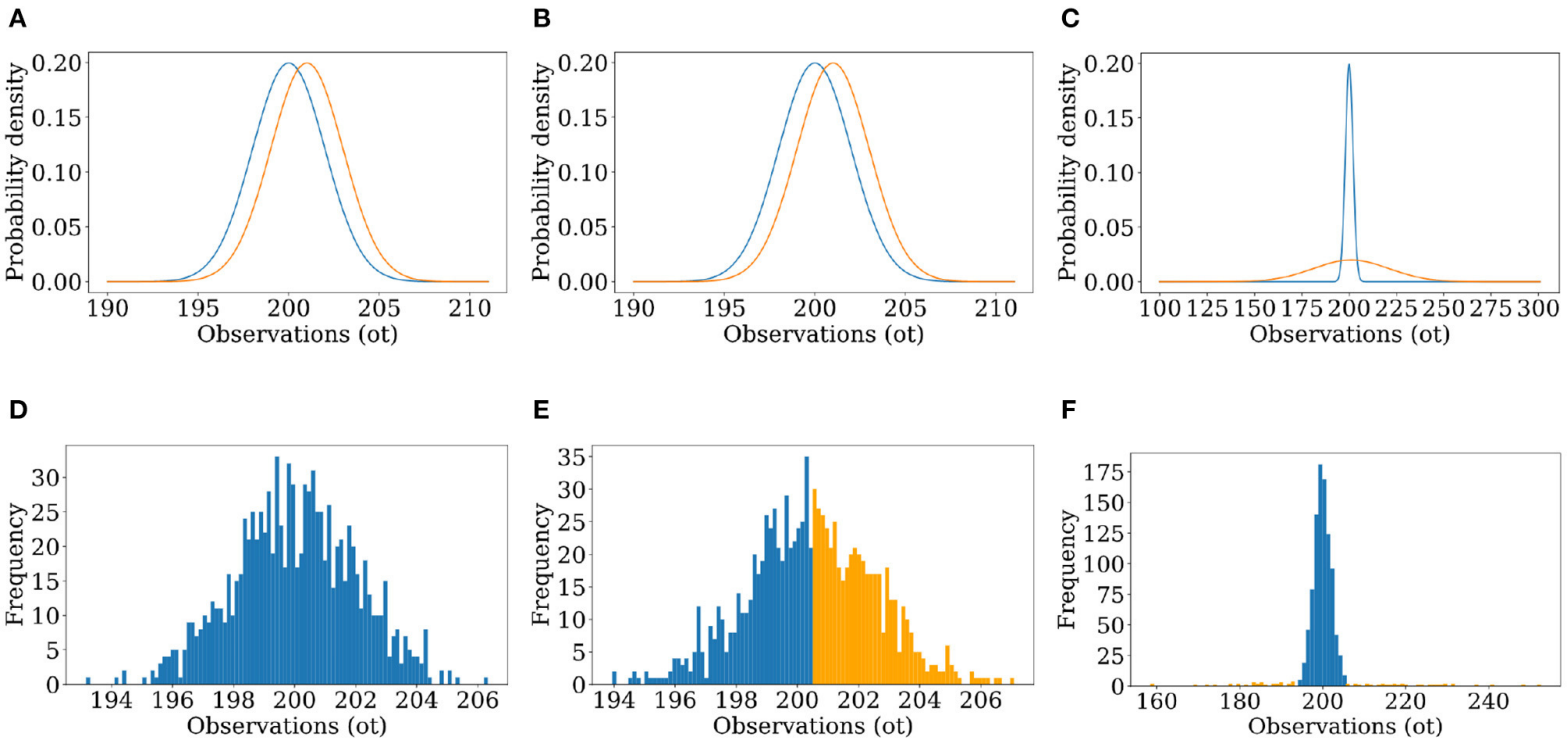
**(A–C)** Graphs of the distributions used to generate the observations of the simulated world and used by the simulated observer to find the likelihood, for unimodal Trials 1–3. **(D–F)** Results of Trials 1–3, showing a histogram of the 1,000 observations of the simulated world colored by the result of classification (blue for classified as real, orange for classified as false).

First, we discuss classification accuracies when *n* = 1, i.e., when the sensory perceptions are low-dimensional and not complex. Figure 3 and Table 1 refer to three representative trials that each illustrate a different phenomenon. In each one, the observer classified 1000 unidimensional observations, and had a perfect model of both accurate judgements and delusions, i.e., $\tilde{\rho }=\rho$ and *f* = *f*′. Table 1 describes the conditions under which the observations were generated, and some quantitative measurements of the success of classification. Figure 3 shows the probability distributions described in Table 1 and the corresponding results of classification, showing how the initial data set is processed by the observer. In trial 1, the real and delusional models overlap significantly, and the frequency of delusions is low; the accuracy rate is correspondingly high. In trial 2, the frequency of delusions has increased, and accuracy has dropped to 56.6%. In trial 3, the frequency of delusions is small, as in trial 1; but the real and delusional models differ substantially. The mere fact that a delusion is highly unlikely and that outliers are highly likely to be delusions is enough to drive the accuracy rate to 97.7%.

**Table 1:** Results of unidimensional classification for three experimental conditions, with perfect observer models.

**Experimental conditions**	**Accuracy rate (%)**	**True positive rate (%)**	**False positive rate (%)**
$\rho_{r}\sim N\left(200,2\right)$, $\rho_{f}\sim N\left(201,2\right)$, *f* = 0.1	91.5	100	100
$\rho_{r}\sim N\left(200,2\right)$, $\rho_{f}\sim N\left(201,2\right)$, *f* = 0.5	56.6	55.4	41.2
$\rho_{r}\sim N\left(200,2\right)$, $\rho_{f}\sim N\left(201,20\right)$, *f* = 0.1	97.7	99.9	22.2

*For the first trial, the classification treated every single one of the 1, 000 observations as true, as seen in Figure 3 (left). The true positive and false positive rates are the possibilities that a real observation is classified as real or that a real observation is classified as false, respectively*.

Figure 3 offers more insight. In trial 2, for an observation below 200.5 (the average of the distributions' means) *L* will always be greater than 1, and the observations will always be classified as real. However, the area under the density curve from *x* = (−∞, 200.5) is nearly the same for both distributions. Even though every observation within that interval will inevitably be classified as real, the likelihood of the generative algorithm producing real or delusional observations in that interval is around the same. After *x* = 200.5, ρ_*f*_ values are now always greater than ρ_*r*_. *L* will always be less than 1, and the observations will all be classed as delusional despite a non-zero probability of them being real. In trial 1, *f*—the frequency of delusion—is small. Multiplying ρ_*r*_ by 1−*f* and ρ_*f*_ by *f* leads to *L* being greater than 1 in all cases. When ρ_*r*_ and ρ_*f*_ are very close together, it is impossible to discriminate between them based on the value of the observations, and so our prior—the frequency of delusion—becomes the main determinant in how we classify observations.

Trial 3 classified 922 of 1, 000 observations as real, with an accuracy rate of 97.7% and a false positive rate of 19.79%. Here, the distributions are more distinct. There is still one interval of overlap where neither ρ_*r*_ nor ρ_*f*_ are negligible, but ρ_*r*_ is always greater. As such, the observations are always classified as real even when there is a chance of them being delusional, contributing to the false positive rate. The distribution ρ_*f*_ is, however, wide enough in spread to produce two large zones where there is minimal overlap between the distributions and where ρ_*r*_ is negligible. Within these areas only false observations are expected to be generated and all are expected to be classified correctly, raising the overall accuracy.

In short, observations are classified as real or delusional based on the interval that they are in. A delusional observation that happens to fall in a “real interval” will always be misclassified as real. An extraordinary delusion can be distinguished from the real, but an ordinary delusion which occupies the same interval cannot.

### 4.3. Rescuing the Accuracy Rate by Increasing Dimensionality

The sometimes poor accuracy rates of the previous subsection can be increased markedly by increasing the dimensionality of the sensory perception. Each element of the vector describing the sensory perception provides independent information about whether or not the perception is real or delusional. Put together, the accumulated evidence more correctly points to the nature of the observation than any one element of the observation vector.

**Figure 4:**
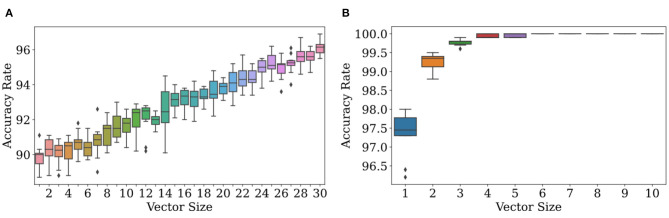
Accuracy increases with the size of the observation vector when its size is ≥ 1. At **(A)**, ϕ_*r*_ = *N*(200, 2), ϕ_*f*_ = *N*(201, 2), and *f* = 0.1. Due to the similarity of the two distributions, the accuracy rate increases slowly. At **(B)**, ϕ_*r*_ = *N*(200, 2), ϕ_*f*_ = *N*(201, 20), and *f* = 0.1. Due to the high variance in ϕ_*f*_ and thus dissimilarity in the two distributions, the accuracy rate increases quickly to nearly 100%.

As observations get more complex, Bayesian classification become more effective at determining which distributions they come from. We see strong numerical evidence of this in Figure 4. Figure 4 (left) displays results of ten trials for each of 30 dimensionalities with the experimental conditions listed in the caption. There is a clear positive correlation between accuracy and dimensionality. In Figure 4 (right), accuracy jumps from 97.5 to 100% at dimensionalities of 5 with high probability, meaning that most trials led to accuracy rates near 100%.

The rate at which scatter in accuracy decreases is extremely dependent on environment. From Figure 4 (right), it appears that scatter in accuracy rate can be made quite small at even *n* = 5 for those environmental conditions; but at *n* = 30 in Figure 4 (left), the scatter is still quite noticeable.

Multidimensional observations allow the classification algorithm to decide on real or delusional with more information than would be afforded by a scalar observation. Suppose *n* = 10. If an observation is just one number, a number drawn from the false distribution can land in an interval where ρ_*r*_ exceeds ρ_*f*_; but out of a set of ten numbers drawn from ρ_*f*_, a majority may be within the interval where ρ_*f*_ exceeds ρ_*r*_. By leveraging the power of more data, we can achieve accuracy rates approaching 100% with probability one by increasing *n*, the dimensionality of sensory perceptions. In real life, sensory perceptions can be of very high dimensionality, and so this analysis is likely more relevant than that of the unidimensional case. A full theoretical analysis is contained in section 4.4 below.

### 4.4. Imperfect Models Can Still Lead to Near-Perfect Accuracy

Until now, we have assumed that the observer's model of the environment is perfectly accurate. We have assumed that the observer's estimation of the prior probability that any given observation is delusional is exactly the same as the environmental variable *f*, and that the observer has also identified the distributions ρ_*r*_ and ρ_*f*_ that observations are drawn from. Five simulations were run in which the observer had an incorrect perception of the prior probability, or an incorrect perception of the mean of one or both distributions. Perhaps surprisingly, under weak conditions, imperfect models can lead to near-perfect accuracy rates.

In section 4.3, we showed that increasing the dimensionality of observations will always raise the accuracy rate of classification. This is only sometimes true when one has an imperfect model. To illustrate this, we focus on five different imperfect models at a range of dimensionalities. See Table 2. There will be two quantities that determine whether or not the accuracy rate increases with increasing dimensionality, μ_*r*_ and μ_*f*_:

(5) $$\begin{array}{r} \mu_{r/f}=n\overset{\infty }{\underset{-\infty }{\int }}\rho_{r/f}\left(o\right)\log\frac{{\tilde{\rho }}_{r/f}\left(o\right)}{{\tilde{\rho }}_{f/r}\left(o\right)}do. \end{array}$$

**Table 2:** Descriptions of the observer's assumptions for each trial, with the true environmental conditions given in Figure 5.

**Environmental Model:ρ_*****r*****_ = ***N***(200, 2)**, **ρ_*****f*****_ = ***N***(201, 2)**, ****f**= 0.1**		
**Observer's models**	$\int_{-\infty }^{\infty }\rho_{r}(o)\ln\frac{{\tilde{\rho }}_{r}(o)}{{\tilde{\rho }}_{f}(o)}do$	$\int_{-\infty }^{\infty }\rho_{f}(o)\ln\frac{{\tilde{\rho }}_{r}(o)}{{\tilde{\rho }}_{f}(o)}do$
${\tilde{\rho }}_{r}=N\left(200,2\right)$, ${\tilde{\rho }}_{f}=N\left(201,2\right)$, *f* = 0.2	0.125	0.125
${\tilde{\rho }}_{r}=N\left(200,2\right)$, ${\tilde{\rho }}_{f}=N\left(201,2\right)$, *f* = 0.9	0.125	0.125
${\tilde{\rho }}_{r}=N\left(196,2\right)$, ${\tilde{\rho }}_{f}=N\left(205,2\right)$, *f* = 0.1	1.125	1.125
${\tilde{\rho }}_{r}=N\left(198,2\right)$, ${\tilde{\rho }}_{f}=N\left(199,2\right)$, *f* = 0.1	−0.375	0.625
${\tilde{\rho }}_{r}=N\left(202,2\right)$, ${\tilde{\rho }}_{f}=N\left(203,2\right)$, *f* = 0.1	0.625	−0.375

*These are accompanied by divergence scores calculated according to Equation (5)*.

As we will show later, there are good theoretical reasons to suspect that negative μ_*r*/*f*_ leads to a less than perfect accuracy rate in the limit of large observation length. But for now, we will show corroborating empirical evidence.

**Figure 5:**
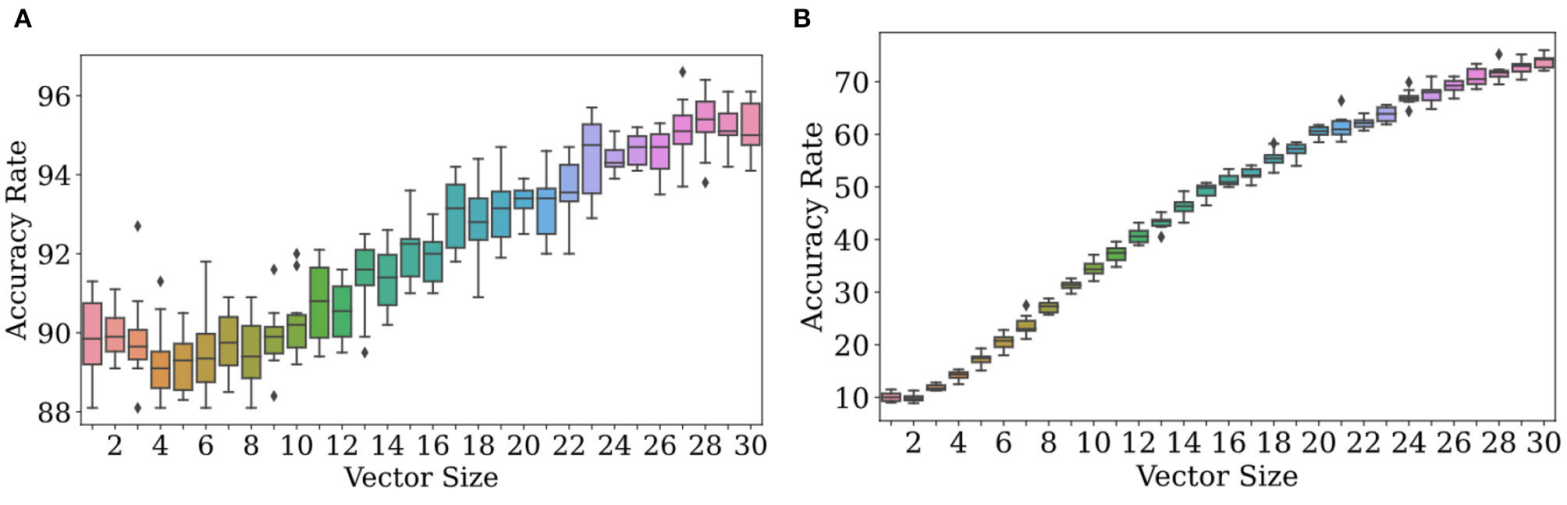
Classification with priors and models that don't match the environmental values can lead to increases in accuracy rate to near 100%. Observations were generated with the environmental model given in Table 2. For classification, the observer used the distributions given in the first two rows of Table 2. **(A,B)** both show results of the trials where the simulated observer has an accurate grasp of the environmental distributions [$\tilde{\rho }$r = N(200, 2), $\tilde{\rho }$f = N(201, 2)] and prior probability *f* of false observations. The prior probability is *f* = 0.2 for the trial whose results are shown in **(A)**, and *f* = 0.9 for the trial whose results are shown in **(B)**.

**Figure 6:**
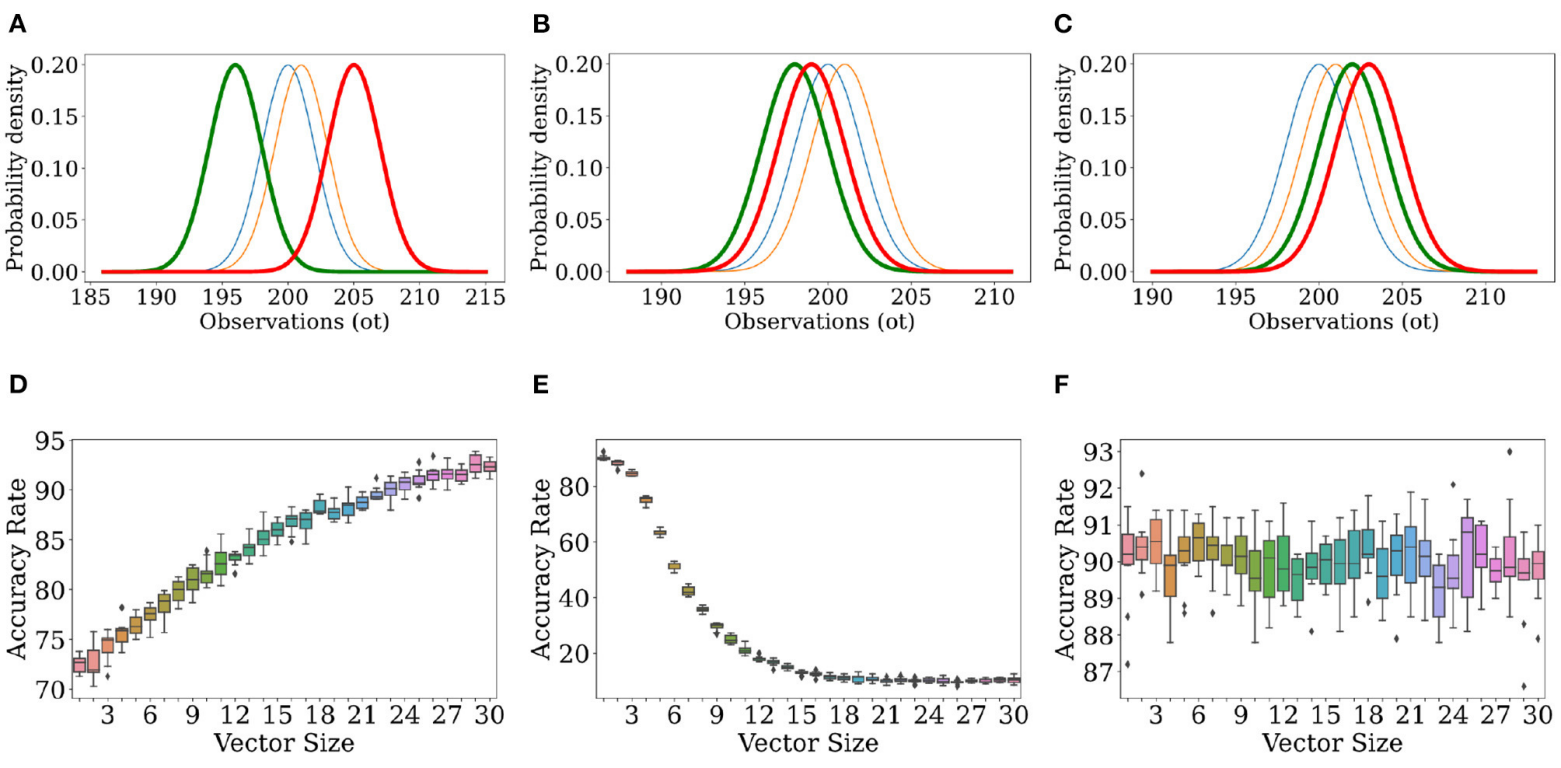
Classification with priors and models that don't match the environmental values can lead to increases or decreases in accuracy rate. Observations were generated with the environmental model given in Table 2. For classification, the observer used the distributions given in the last three rows of Table 2. The top row shows ρ_*f*_ (), ρ_*r*_ (), ${\tilde{\rho }}_{f}$ (), and ${\tilde{\rho }}_{r}$ (), while the bottom row shows the corresponding accuracy rate as a function of observation length. The environmental distributions and observer-constructed distributions given in Rows 3–5 of Table 2 are illustrated in **(A)** (Row 3), **(B)** (Row 4), and **(C)** (Row 5). **(D)** describes the results for the trial using the conditions shown in **(A)**, **(E)** shows the results from **(B)**, **(F)** shows the results from **(C)**.

In Trials 1, 2, and 3, as illustrated in Figure 5 (left, right), increasing the dimensionality increases the accuracy rate. In all of these trials, both μ_*r*_ (the mean of the log-scaled summation of likelihood ratios for classification of real observations in Equation 5) and μ_*f*_ (the equivalent for classification of fake observations) are positive as seen in Table 2. However, in Trials 4 and 5, as illustrated in Figure 6 (middle, right), increasing dimensionality does not increase accuracy. For Trial 4, the accuracy actually decreases; and for Trial 5, the accuracy remains, with some scatter, fixed around 1−*f* in a manner reminiscent of the trials with unidimensional observations. For these two trials, either μ_*r*_ or μ_*f*_ is negative, and accuracy tends to decrease with increasing dimensionality.

Let's focus on Trials 3−5 so that we can, in detail, understand the effects of a mismatched model.

In Trial 3, the observer's perception (${\tilde{\rho }}_{r}$) of ρ_*r*_ has a mean (196) two standard deviations below the environmental value (200), and the observer's perception (${\tilde{\rho }}_{f}$) of ρ_*f*_ has a mean (205) two standard deviations above the environmental value (201). Figure 6A illustrates the consequence of this: due to the minimal overlap between these distributions, any environmental observation judged to have been likely to be drawn from ${\tilde{\rho }}_{r}$ is judged as impossible to draw from ${\tilde{\rho }}_{f}$, and vice versa. Hence, μ_*r*_ and μ_*f*_ are larger in Trial 3 as compared to Trials 1 and 2. In other words, because ${\tilde{\rho }}_{r}$ is so much more likely to be low when ${\tilde{\rho }}_{f}$ is high and vice versa, the likelihood ratio used to calculate μ_*r*/*f*_ is more extreme, leading to larger values for μ_*r*/*f*_. However, μ_*r*/*f*_ are both still positive for Trial 3, and as in Trials 1 and 2, the accuracy rate is rescued. So long as the observation vectors are filled in randomly, larger vectors may (if real) be filled in with more values which are likelier to be from ρ_*r*_ than ρ_*f*_, and (if false) be filled in with more values which are likelier to be from ${\tilde{\rho }}_{r}$ rather than ${\tilde{\rho }}_{f}$, and so there is a basis for correct decision-making that is improved upon by increasing dimensionality.

In Trial 4, the observer's perception ${\tilde{\rho }}_{f}$ actually overlaps more with ρ_*r*_ than ${\tilde{\rho }}_{r}$ does. When for a real observation ${\tilde{\rho }}_{r}$ is consistently lower than ${\tilde{\rho }}_{f}$, μ_*r*_ is consistently negative because the ratio within the logarithm is consistently less than 1. This removes the basis for correct decision-making present in Trial 3. There instead arises a tendency to classify everything as fake, resulting in a decreasing accuracy with a horizontal asymptote around *f* = 0.1, the prior probability that an observation was fake to begin with. In other words, the observer classifies everything as fake, and is right around 10% of the time because the environment is supposed to make around 10% of the observations fake in every case.

In Trial 5, ${\tilde{\rho }}_{r}$ overlaps more with ρ_*f*_ than ${\tilde{\rho }}_{f}$, resulting in a tendency to classify everything as real, which is right 90% of the time (1−*f* = 0.9). Here, μ_*f*_ is negative, and for a similar reason as before.

A straightforward theoretical argument explains these findings, and further illustrates their robustness to simulation parameters. Consider classifying just one observation *o*. We can rewrite

(6) $$\begin{array}{r} L=\frac{1-f^{\prime }}{f^{\prime }}\exp\left(\overset{n}{\underset{i=1}{\sum }}\log\frac{{\tilde{\rho }}_{r}\left(o_{i}\right)}{{\tilde{\rho }}_{f}\left(o_{i}\right)}\right). \end{array}$$

According to the Central Limit Theorem (Pishro-Nik, 2016), the summation in the exponent tends toward a normally distributed random variable in the large *n* limit. If the observation is real/fake, then the mean of this normally distributed random variable is given by

(7) $$\begin{array}{r} \mu_{r/f}=n\overset{\infty }{\underset{-\infty }{\int }}\rho_{r/f}\left(o\right)\log\frac{{\tilde{\rho }}_{r/f}\left(o\right)}{{\tilde{\rho }}_{f/r}\left(o\right)}do \end{array}$$

as shown earlier and the variance is given by

(8) $$\begin{array}{l} \sigma_{r/f}^{2}=n(\int_{-\infty }^{\infty }\rho_{r/f}(o)\log^{2}\frac{{\tilde{\rho }}_{r/f}(o)}{{\tilde{\rho }}_{f/r}(o)}do\\ \;-{\left(\int_{-\infty }^{\infty }\rho_{r/f}(o)\log\frac{{\tilde{\rho }}_{r/f}(o)}{{\tilde{\rho }}_{f/r}(o)}do\right)}^{2}). \end{array}$$

The accuracy of the classification algorithm, averaged over many observations, is

(9) $$\begin{array}{rcl} A & = & \left(1-f\right)P\left(L>1|R\right)+fP\left(L<1|F\right) \end{array}$$

where (1 − *f*) represents the probability that the observation is real, *P*(*L* > 1|*R*) represents the probability that we classify the observation as real given that the observation is real, *f* represents the probability that the observation is fake, and *P*(*L* < 1|*F*) represents the probability that we classify the observation as fake given that the observation is fake. If *Z* represents the normally distributed random variable, then

(10) $$\begin{array}{r} P\left(L>1|R\right)=P\left(\frac{1-f^{\prime }}{f^{\prime }}e^{Z}>1\right)=P\left(Z>\log\frac{f^{\prime }}{1-f^{\prime }}\right) \end{array}$$

From this we can conclude that

(11) $$\begin{array}{ccc} P\left(L>1|R\right) & = & \frac{1}{2}erfc\left(\frac{\log\frac{f^{\prime }}{1-f^{\prime }}-n\overset{\infty }{\underset{-\infty }{\int }}\rho_{r}\left(o\right)\log\frac{{\tilde{\rho }}_{r}\left(o\right)}{{\tilde{\rho }}_{f}\left(o\right)}do}{\sqrt{2n}{\left(\overset{\infty }{\underset{-\infty }{\int }}\rho_{r}\left(o\right)\log^{2}\frac{{\tilde{\rho }}_{r}\left(o\right)}{{\tilde{\rho }}_{f}\left(o\right)}do-{\left(\overset{\infty }{\underset{-\infty }{\int }}\rho_{r}\left(o\right)\log\frac{{\tilde{\rho }}_{r}\left(o\right)}{{\tilde{\rho }}_{f}\left(o\right)}do\right)}^{2}\right)}^{1/2}}\right)\;\left(11\right) \end{array}$$

and

(12) $$\begin{array}{ccc} P\left(L<1|F\right) & = & \frac{1}{2}erfc\left(\frac{\log\frac{1-f^{\prime }}{f^{\prime }}-n\overset{\infty }{\underset{-\infty }{\int }}\rho_{f}\left(o\right)\log\frac{{\tilde{\rho }}_{f}\left(o\right)}{{\tilde{\rho }}_{r}\left(o\right)}do}{\sqrt{2n}{\left(\overset{\infty }{\underset{-\infty }{\int }}\rho_{f}\left(o\right)\log^{2}\frac{{\tilde{\rho }}_{f}\left(o\right)}{{\tilde{\rho }}_{r}\left(o\right)}do-{\left(\overset{\infty }{\underset{-\infty }{\int }}\rho_{f}\left(o\right)\log\frac{{\tilde{\rho }}_{f}\left(o\right)}{{\tilde{\rho }}_{r}\left(o\right)}do\right)}^{2}\right)}^{1/2}}\right)\;\left(12\right) \end{array}$$

where erfc is defined in Pishiro-Nik (Pishro-Nik, 2016). Altogether, we now have a closed-form expression for the average accuracy in the large *n* limit. Suppose that $f<\frac{1}{2}$, so that delusions are rare. Some analysis in Mathematica 12 shows that, in the large *n* limit, accuracy approaches 1 with correction factors of order $\frac{1}{n^{3/2}}$ as long as $\overset{\infty }{\underset{-\infty }{\int }}\rho_{r/f}\left(o\right)\log\frac{{\tilde{\rho }}_{r/f}\left(o\right)}{{\tilde{\rho }}_{f/r}\left(o\right)}do>0$. This inequality is guaranteed if the model is completely correct, $\tilde{\rho }=\rho$, as then the left-hand side of the inequality is the Kullback-Leibler divergence (MacKay, 2003), between ρ_*r*_ and ρ_*f*_ or vice versa– a non-negative measure of distance between the two distributions– therefore explaining the results in Figure 6.

In Figures 5, 6, we see varying rates of convergence to final values of accurate rate. The estimation of the frequency *f*′ of delusions does not affect convergence properties strongly. Rather, the rate at which the accuracy approaches 1 is affected by a complicated function of ρ and $\tilde{\rho }$, indicating that one's model and the true nature of the world are both fundamental to how well one's classification algorithm work. In particular, the rate at which accuracy increases or decreases with increasing dimensionality is governed by parameters σ_*r*/*f*_. Note from Equation (9) and the equations that follow that imperfect information about the frequency of delusions is essentially unimportant in determining both the accuracy rate at large dimensionalities and the rate of increase in accuracy rate with increasing dimensionality.

The analysis above assumes a large *n*, such that the scatter in *L* is minimized. But, for small enough *n*, the scatter in *L* might be substantial, leading to a large scatter in accuracy. For example, perhaps a majority of the vector components of the sensory perception aren't drawn from the right interval or that the likelihoods are so evenly matched that the prior-ratio is able to overwhelm the difference as seen in Table 1.

## 5. Discussion

We believe that cognitive approaches to schizophrenia therapy are a healthier approach to making judgements, or dealing with fixed ideas, as their goal, but accept that since our approach is so dependent on CBT for theoretical support it is worth looking at some of the criticisms of it as well. Since the 1990s, the cognitive model of auditory hallucinations introduced by Chadwick and Birchwood (Chadwick and Birchwood, 1994) has suggested that a person's (potentially delusional) beliefs about the identity, powers, and intentions of their AVH, as well as their own ability to control aspects of these voices, are predictors for the distress, depression, or problematic responses (for example, compliance with a hallucination that manifests as a command to do something) these hallucinations cause (Thomas et al., 2014). A study with small samples of individuals from India, Ghana, and the United States found that while the Indians perceived their AVH as the voices of elders telling them to do simple things and the Ghanaians perceived their AVH as the voice of God, the Americans heard unidentifiable voices which discussed and called for violence (Luhrmann et al., 2015). Meanwhile, people with low self-esteem and feelings of being unsafe may hear voices which remind them of experiences of being unsafe (Taylor et al., 2020), and may also perceive new people as threatening on the basis of little evidence (Garrett et al., 2019).

To address the interlinked challenges of schizophrenia, cognitive behavioral therapy for psychosis (CBTp) places its focus on helping people think about their symptoms from a more helpful perspective, or cope with their symptoms in a more effective manner (Thomas et al., 2014). Individualized case formulation is the distinguishing feature of CBTp, because several aspects of schizophrenia can vary between individuals (symptom presentation and severity, hospitalization, self-esteem, family support, response to medication) with independent and interlinked effects on their chances of recovery (Turkington et al., 2003). The particulars of CBT depend on mutual agreement between each individual patient and their therapist, but may consist of psychoeducation (conventional education about a disorder and its symptoms), identification and review of beliefs, exercises to build awareness of emotional states or other triggers for psychotic symptoms, and exercises to seek out more positive emotional states (Freeman et al., 2015).

A criticism of CBT has been its tendency to exist as a “black box” which is hard to evaluate with conventional randomized control trials– because CBT strives to be as individualized as possible, mixing in different exercises or even different theoretical underpinnings, there is a limit to how much can be said about its general effectiveness in reducing the severity of specific or overall psychosis-related symptoms, or about the effectiveness of the exercises it contains (Thomas, 2015). A subset of CBTp known as CBTv (CBT for voices), which retains the emphasis on challenging patients' beliefs about themselves but focuses specifically on those beliefs about voice omnipotence and personal submission that make coping with AVH especially difficult, has been promoted– but there is not yet sufficient evidence to conclude that it lives up to its promise of being just as effective as (or more effective than) CBTp while requiring less clinician expertise, which would ideally allow more clinicians to participate in delivering this service (Hazell et al., 2018). In addition, anywhere from 80% of individuals with schizophrenia lack insight into, or awareness of, their illness and symptoms (Joseph et al., 2015). In one case study, a patient was aware of his symptoms, but insisted that there had to be some physiological cause with them, some defect in his body, rather than a mental illness (Bastiaens and Agarkar, 2014). Several studies have outlined how, when uncertain about the source of a perceived event, individuals with schizophrenia will prefer to attribute it to an external source (Engh et al., 2010). The effect is a lack of faith in treatment, and consequently a lack of adherence to it- since they do not fundamentally believe themselves to be mentally ill even if informed of that fact by others, they will consciously refuse or merely forget to take medicine doses and other treatments, which may lead to relapse (Bitter et al., 2015). This study takes anosognosia (another term for “lack of insight”) as an indication that individuals with schizophrenia cannot be expected to agree with others on the cause of their symptoms, even when they acknowledge the existence of those symptoms (Engh et al., 2010). Therefore, CBT and similar therapies are successful to the extent that they help patients build an understanding of when and how they come to initiate negative emotional states like worry or fear, review harmful assumptions about these states or the beliefs that justify them, and learn to plan around them or deal with them (Freeman et al., 2010).

However, achieving true self-awareness may not be necessary. So long as an individual can acknowledge that their delusions at least have unusual or uninvited qualities, which even patients with grandiose delusions of divinity are capable of doing (Isham et al., 2019), and keep track of the way they deviate from what is expected of the real world, there can be a way for them to discriminate between delusions and non-delusional observations no matter what their attitudes on the delusion's truth or falsity are (Beck and Rector, 2005). This is all in the general tradition of cognitive therapy going back to Aaron Beck's identification of the conserved nature of depressive cognition, and his development of a therapeutic approach that encouraged patients to recognize how different situations could lead to similar depressive thoughts, to anticipate the thoughts they will have on entering a situation, and to prepare in advance against them (McKenna, 2017).

Although the question of how effective a treatment CBT is remains an enigma, we believe the results of our demonstration of Bayesian inference in classification suggest that a person, as long as efforts are taken to develop their model of the world, can find consciously adopting Bayesian reasoning helpful for dealing with delusions. Figure 1 illustrates a simplified scheme for implementing this, by encouraging a patient to challenge their initial judgements, take in more observational information, and revise their assumptions. If people can be made to imagine the real and unreal as overlapping ranges of possibilities, Bayesian inference can provide a method for figuring out which of these locations any given observation comes from.

It should also be clarified that in recommending a technique to aid conscious thinking, this study makes no claims about the fundamental mechanisms on which human cognition operates. Advancements in artificial intelligence have influenced theories of human cognition, though some approaches to AI are better at imitating human abilities than others. For example, people and “neural networks” can both be trained to recognize handwritten letters and numbers with high accuracy, but while people can recognize handwritten characters after seeing only a single example (and also store this information as a flexible mental concept which is open to future changes), the MNIST data set for recognizing digits contains 60, 000 images (6, 000 for each digit 0−9) people can recognize handwritten characters after seeing only a single example (Lake et al., 2017). To imitate the results as well as the starting conditions of human cognition, a machine must make inferences that go beyond the data available to the machine for the task at hand. One way to do this is Bayesian modeling with strong priors, in which a computer can start with limited data but continually revise an assumption over the course of making a new decision (Lake et al., 2017). The idea that human brains use rules as precise and systematic as those of mathematical models has been challenged, but there is not yet sufficient evidence to rule out the idea that the brain works like a computer and the success of probabilistic algorithms in AI applications has ensured their popularity in theories of cognition (Gershman et al., 2015). Although the literature on the relevance of Bayesianism to psychology often frames it as a way to explain brain activity, this study does not have the same goal; it remains agnostic on the fundamental nature of the brain or on what normative principles can explain schizophrenia, and devises a proof of concept for an acquired and imitable method.

## 6. Conclusion

In this study, we try to study reality-testing from a computational perspective. Patients with schizophrenia may be able to decide whether specific observations on their environment can be relied on or not, on the basis of prior estimates of the probability that any observation is real or delusional and the likelihood that a specific observation belongs in either category based on its attributes (which may be characteristic of one or both categories). This study assumes that this decision takes the form of comparing the results of two calculations: the probability that the observation is real, and the probability that the observation is false. Classification proceeds by comparing the ratio of these probabilities to 1.

This study concludes that when an observation is low-dimensional (simple) and are just as likely to have emerged from either distribution, the prior probabilities of any observation being real or fake are of greater importance to the final decision than the attributes of the observation, and so a Bayesian thinker will either doubt everything or doubt nothing within particular intervals. However, when an observation is high-dimensional (complex), classification accuracy tends to 100% as long as the patient's model of the world is not drastically inaccurate.

The principle that noting more attributes about an object or event allows for a better understanding of it is common sense, but in this study it finds mathematical expression, which allows this principle to potentially inform the design of therapies. This study also shows an important fact about Bayes' theorem: an optimal decision informed by limited information can be very different from an optimal decision informed by more comprehensive input.

## Data Availability Statement

The original contributions presented in the study are included in the article/supplementary material, further inquiries can be directed to the corresponding author/s.

## Author Contributions

SM conceived of the idea and developed the theory. BA and DL wrote the paper, performed the computations, and performed all background research. All authors contributed to the article and approved the submitted version.

## Conflict of Interest

The authors declare that the research was conducted in the absence of any commercial or financial relationships that could be construed as a potential conflict of interest.

## Publisher's Note

All claims expressed in this article are solely those of the authors and do not necessarily represent those of their affiliated organizations, or those of the publisher, the editors and the reviewers. Any product that may be evaluated in this article, or claim that may be made by its manufacturer, is not guaranteed or endorsed by the publisher.
